# A national survey of digital health company experiences with electronic health record application programming interfaces

**DOI:** 10.1093/jamia/ocae006

**Published:** 2024-01-27

**Authors:** Wesley Barker, Natalya Maisel, Catherine E Strawley, Grace K Israelit, Julia Adler-Milstein, Benjamin Rosner

**Affiliations:** Office of the National Coordinator for Health Information Technology, US Department of Health and Human Services, Washington, DC 20201, United States; Division of Clinical Informatics & Digital Transformation, Department of Medicine, University of California San Francisco, San Francisco, CA 94143, United States; Office of the National Coordinator for Health Information Technology, US Department of Health and Human Services, Washington, DC 20201, United States; Division of Clinical Informatics & Digital Transformation, Department of Medicine, University of California San Francisco, San Francisco, CA 94143, United States; Division of Clinical Informatics & Digital Transformation, Department of Medicine, University of California San Francisco, San Francisco, CA 94143, United States; Division of Clinical Informatics & Digital Transformation, Department of Medicine, University of California San Francisco, San Francisco, CA 94143, United States

**Keywords:** electronic health record, application programming interface, digital health, industry

## Abstract

**Objectives:**

This study sought to capture current digital health company experiences integrating with electronic health records (EHRs), given new federally regulated standards-based application programming interface (API) policies.

**Materials and methods:**

We developed and fielded a survey among companies that develop solutions enabling human interaction with an EHR API. The survey was developed by the University of California San Francisco in collaboration with the Office of the National Coordinator for Health Information Technology, the California Health Care Foundation, and ScaleHealth. The instrument contained questions pertaining to experiences with API integrations, barriers faced during API integrations, and API-relevant policy efforts.

**Results:**

About 73% of companies reported current or previous use of a standards-based EHR API in production. About 57% of respondents indicated using both standards-based and proprietary APIs to integrate with an EHR, and 24% worked about equally with both APIs. Most companies reported use of the Fast Healthcare Interoperability Resources standard. Companies reported that standards-based APIs required on average less burden than proprietary APIs to establish and maintain. However, companies face barriers to adopting standards-based APIs, including high fees, lack of realistic clinical testing data, and lack of data elements of interest or value.

**Discussion:**

The industry is moving toward the use of standardized APIs to streamline data exchange, with a majority of digital health companies using standards-based APIs to integrate with EHRs. However, barriers persist.

**Conclusion:**

A large portion of digital health companies use standards-based APIs to interoperate with EHRs. Continuing to improve the resources for digital health companies to find, test, connect, and use these APIs “without special effort” will be crucial to ensure future technology robustness and durability.

## Background and significance

Over the past decade, and increasingly over the past few years, electronic health record (EHR) developers have implemented application programming interfaces (APIs) in response to the need to open their systems to third-party applications. In particular, as called for in the 2014 JASON report, *A Robust Health Data Infrastructure*, and Office of the National Coordinator for Health IT (ONC)-funded work led by Substitutable Medical Apps & Reusable Technology (SMART) and the Argonaut Project, standards-based APIs were essential to allow scalable integrations.^1–3^ Standards-based APIs harmonize connections across different EHRs and facilitate third-party software integration, thereby improving interoperability by enabling streamlined and secure data exchange.^4^ The progress of these efforts and maturity of APIs set the stage for federal regulations, implementing provisions of the 21st Century Cures Act, that made standards-based APIs the default method for third-party applications to access and exchange patient electronic health information from EHRs certified to the criteria and standards adopted by the US Department of Health and Human Services (HHS).^5^ In particular, these regulations, finalized in 2020, adopted the Health Level Seven (HL7) Fast Healthcare Interoperability Resources (FHIR) data exchange standard to enable third-party app developers to connect to certified EHRs.^6^ Certified health IT developers were required to implement these APIs by 2022.

While the intent of these efforts—to improve interoperability—is clear, to what extent and for what use cases these standards-based APIs succeed in doing so is less clear. Historically, a 2016 survey of digital health companies found that a substantial number had attempted integrations with EHRs but encountered barriers, including a lack of developer support from EHR vendors, overall difficulty partnering with EHRs, and high associated costs.^7^ A follow-up survey in 2018 found progress in companies’ abilities to integrate with EHRs through APIs, though challenges still remained.^8^ Other studies have examined the availability of certain technologies integrated with EHRs (ie, capturing what was successfully integrated) and the overall robustness and durability of individual EHR company’s resources for third-party developers.^4^^,^^9^ However, both prior digital health company surveys took place before the 2022 implementation deadline and included responses from less than 100 companies that had ever integrated their technology with an EHR. We therefore undertook an updated survey of these companies to capture the early impact of these regulations. We specifically sought to assess 3 dimensions. First, it is important to evaluate the use of standards-based versus proprietary EHR APIs to get a snapshot of national progress toward streamlined health data exchange between EHRs and third-party applications. Second, understanding company experiences integrating with specific EHR vendors (eg, Epic, Cerner) as well as the total number of vendors provides insight into the extent of interoperability of digital health company products. Third, it is critical to understand enablers of and barriers to EHR integration to inform ongoing policy and industry efforts to advance APIs and EHR integration.

## Objective

This study sought to capture current digital health company experiences integrating with EHRs, now that new federally regulated standards-based API policies are in place and being implemented by EHR vendors. The survey covered company experience with EHR API integration, barriers to EHR integrations, and API policy and advancement efforts to ensure a robust perspective from digital health companies who are the primary consumers of these EHR APIs. These perspectives directly inform both policymakers and industry stakeholders on how to deliver next-generation technology solutions to health care providers and consumers. In particular, results will serve to guide the Department of Health and Human Services (HHS) on where ongoing policymaking may be needed to fulfill the intent of the 21st Century Cures Act. Results will also serve to guide EHR vendors and third-party software companies on the prevalence of ecosystem pain points that could lend themselves to private-sector solutions.

## Methods

### Sample data sources

A list of digital health companies to survey was compiled from a variety of data sources. The majority of companies (*n* = 605) came from a data scraping methodology developed by Barker & Johnson, which pulled company data from public app galleries for EHR-integrated solutions available from 1uphealth, Allscripts, athenahealth, CMS Bluebutton, CARIN Alliance, Cerner Corporation, eMDs, Epic Systems Corporation, Greenway, NextGen, and SMART.^9^ Scraped data included the company name, the number of app galleries in which a company was found; the number of unique apps, names of apps, and functional app categories associated with a company; the targeted users of the company’s technology, and the company’s webpage. Since this method only identified companies that had been successful in integrating at least 1 app with an EHR or EHR-associated platform, we sought to capture a broader set of companies that may have attempted EHR integrations but have not been successful. We supplemented the preliminary list by pulling companies from: (1) a 2020 CB Insights Report titled “The digital health startups transforming the future of healthcare,” (*n* = 20),^10^ (2) an analysis of relied upon software reported through the ONC Health IT Certification Program (*n* = 9), and (3) members of a national expert advisory board convened to support this project (*n* = 110) (see Table S1 for the list of members).

#### Inclusion criteria

Once we developed the list of companies across these 4 sources, we sought to limit it to those that develop solutions that enable human interaction with an API, such as provider-facing apps that access clinical data, either alone or in combination with non-clinical data, as well as patient-facing apps that access clinical or non-clinical data. These criteria exclude companies that solely make solutions that do not enable human interaction with an API, such as external databases or networks that connect to EHRs, apps that enable integration between 2 EHR systems, and provider-facing apps that do not access clinical data—given that these use cases are not the focus of federal regulations and face a different set of challenges. We also sought to exclude companies that make solutions that do not connect to an EHR (primarily those sourced from the CB Insights Report), as well as EHR vendors themselves.

To apply our inclusion criteria, we leveraged the app categories from the data scraping methodology. Companies and apps that were categorized as “clinical use” or “patient care” were included, while companies and apps that were categorized as “administrative” only were excluded. Companies and apps that were categorized as “patient engagement” were manually reviewed to determine inclusion. Manual review primarily involved accessing the app developer’s website or reviewing marketing materials obtained from the online marketplace or gallery to learn more about the app and its intended use. If it was determined that an app’s patient engagement function allowed access to patient records and clinical data, the company was included. For the remaining companies—those that did not have information on their app category, either because they had missing data or were not sourced using the scraping methodology—we first relied on data from the Apple and Google app stores to identify the app’s category. Among apps that could be found in the Apple or Google app stores, those categorized as “medical” were included in our sample, while those categorized as “health and fitness” were excluded. Apps that could not be found in the Apple or Google app stores were manually reviewed by evaluating the marketing materials on the app developer’s website to determine if they met inclusion criteria. This resulted in a final sampling frame of 704 companies.

### Survey development

To capture the current state of progress and challenges that digital health companies face when integrating tools with EHRs, we developed and fielded a survey. The survey instrument was developed by the University of California San Francisco (UCSF) in collaboration with ONC, the California Health Care Foundation, and ScaleHealth (a healthcare solutions marketplace). It was refined based on feedback from the expert advisory board. The survey had 3 sections: (1) Experiences with API integrations, (2) barriers faced during API integrations, and (3) API-relevant policy efforts. The survey was pilot tested with 5 companies and then refined based on feedback. The final instrument can be found in the Supplementary Material.

### Survey administration

Contact information for a target respondent at each company was sourced by ScaleHealth. The survey was distributed via the survey software Qualtrics and was fielded from June to November 2022.

To maximize the response rate, we employed a variety of outreach strategies. These included individual emails not only from UCSF but also from ScaleHealth, our expert advisors, and together. Health to target companies with whom they had existing relationships. We also posted the survey link and information to a variety of message boards, online forums, and listservs (which resulted in capturing 9 additional companies not in our original sampling frame that met inclusion criteria), increasing our total sample to 713. These boards, forums, and listservs included Health Tech Nerds, the American Medical Association Innovation Network, HIMSS Accelerate, ScaleHealth email listservs, the Society of Physician Entrepreneurs LinkedIn group, and the CARIN Alliance email listserv. Lastly, we printed business cards with a QR code link to the survey and distributed them to companies at the 2022 HLTH Conference. We followed-up with non-respondents up to 15 times over the course of survey administration. Incentives to participate in the survey included listing participating companies on public and peer-reviewed reports, providing a copy of the reports to respondents, and inviting respondents to a special session hosted by ONC during which the results and insights from the findings will be shared.

### Analysis

We conducted a set of descriptive analyses based on survey responses. First, we assessed the organizational demographics of the sample, including company relationship with protected heath information (eg, healthcare provider or other covered entity), primary application domain(s), and 2 proxies for size/maturity: company development stage and number of full time equivalent (FTE) staff working on products that integrate with commercial EHRs.

Our first set of analyses sought to capture use of standards-based versus proprietary APIs. We used survey questions that captured company status of integrations with EHRs via proprietary APIs, standards-based APIs, and third-party integration service (eg, Redox). For each integration type, companies were given the following response options: “Yes, in production (currently or previously),” “Yes, in process but not in production,” “Yes, but stopped (incomplete),” or “No.”

We then measured the relationship between the use of standards-based and proprietary APIs by calculating the percent of companies that use 1 type only (standards-based or proprietary), both types, and neither type. We also examined the relative use of proprietary and standards-based EHR APIs for companies that reported using both types by measuring the percent of respondents that reported using each API predominantly, mostly, or equally. Finally, within each of the groups, we calculated the percent of companies that reported using FHIR at all and the percent that used FHIR “extensively” to assess differences between companies’ use of FHIR in their apps across types of EHR API integrations. As FHIR represents the leading industry data standard for RESTful API-based data exchange, it is important to measure how companies’ adoption and use of the standard associates with the types of APIs they used to integrate with EHRs.

Our second set of analyses focused on experiences integrating with specific EHR vendors (eg, Epic, Cerner) as well as the total number of vendors. Through these analyses, we sought to assess the share of companies that integrate with specific EHRs and how adoption of standards-based APIs varies across companies that integrate with 1 or more EHR vendors. Specifically, we calculated the percent of companies that had a successful integration or 1 underway with an EHR. We then stratified the use of FHIR by the number of vendors with which a company integrated (1 vendor, 2-3 vendors, 4+ vendors) and calculated the percent of companies that reported using FHIR at all and the percent that reported using FHIR “extensively” to assess whether companies integrating with more than 1 EHR had higher rates of FHIR use. The core impetus for standardizing API-based exchange is to facilitate app and software integrations across multiple EHRs. We evaluated FHIR use this way because it is important to understand whether FHIR adoption by companies in their products correlates with the number of EHRs with which they integrate.

Our third set of analyses focused on enablers and barriers. First, we calculated the percent of companies that endorsed different dimensions of APIs as “moderately critical” or “critical to a great extent” to the company’s ability to work successfully with EHR APIs. These listed dimensions on the survey included: technical performance, breadth of data elements, and cost. We then calculated the top 10 most prevalent barriers reported by companies as “substantial” barriers to integration from a closed list of 20 barriers. We also compared the effort to establish and maintain proprietary and standards-based APIs to show how reported barriers may differently impact companies’ abilities to establish versus maintain EHR integrations. Finally, we examined open-ended responses to the questions of (1) high-priority clinical data types for future federally regulated availability and (2) future directions for policy efforts in promoting or enforcing access to data. We performed a text analysis of the free-text responses and report the 5 most common responses (grouped by key terms and themes) for each of the questions.

Sample sizes for each measure varied based on item non-response and skip logic (eg, if a company had no API-based EHR integrations, the survey programming logic had them skip many questions on the survey). Missing data were excluded from reported percentages. We conducted a non-response bias analysis to compare company characteristics between respondents and non-respondents. We did not do non-response weighting for reported statistics.

## Results

Of the 713 digital health companies on our final list, 125 companies completed the survey and 16 were considered sufficient partial completers (defined as completing through the questions on effort/resources to establish and maintain integrations with EHR vendors), for a response rate of 20%. A summary of respondent characteristics is included in Table 1.

**Table 1.** Characteristics of digital health company survey respondents.

	Count	Percent (%)
Companies’ relationships with protected health information (PHI)^a^ (*N* = 140)		
Healthcare provider or other covered entity	15	11
Business associate of a covered entity	107	76
Access PHI through consumers outside business associate or covered entity	19	14
Other	8	6
Primary application domain(s)^a^ (*N* = 123^b^)		
Administrative (eg, scheduling, billing, check-in)	46	37
Care delivery, not limited to treatment	102	83
Clinical research	33	27
Patient access and management of health record data	57	46
Population health	51	41
Public health	14	11
Other	13	11
Company development stage (*N* = 122^b^)		
Incubation (pre-seed, seed)	13	11
Early stage (series A and B)	47	39
Development, growth (series C or later)	35	29
Public or acquired by a public company	9	7
Other or not applicable	18	15
Number of FTE staff working on products that integrate with commercial EHRs (*N* = 123^b^)		
1-10	45	37
11-50	47	38
51-100	18	15
101-250	5	4
251-500	5	4
More than 500	3	2

a Groups are not mutually exclusive.

b Denominator differs due to survey question skip logic. Characteristic was collected only from respondents who reported an “in production” or “in process” integration with a commercial EHR.

### Use of standards-based and proprietary APIs

**Figure 1.**
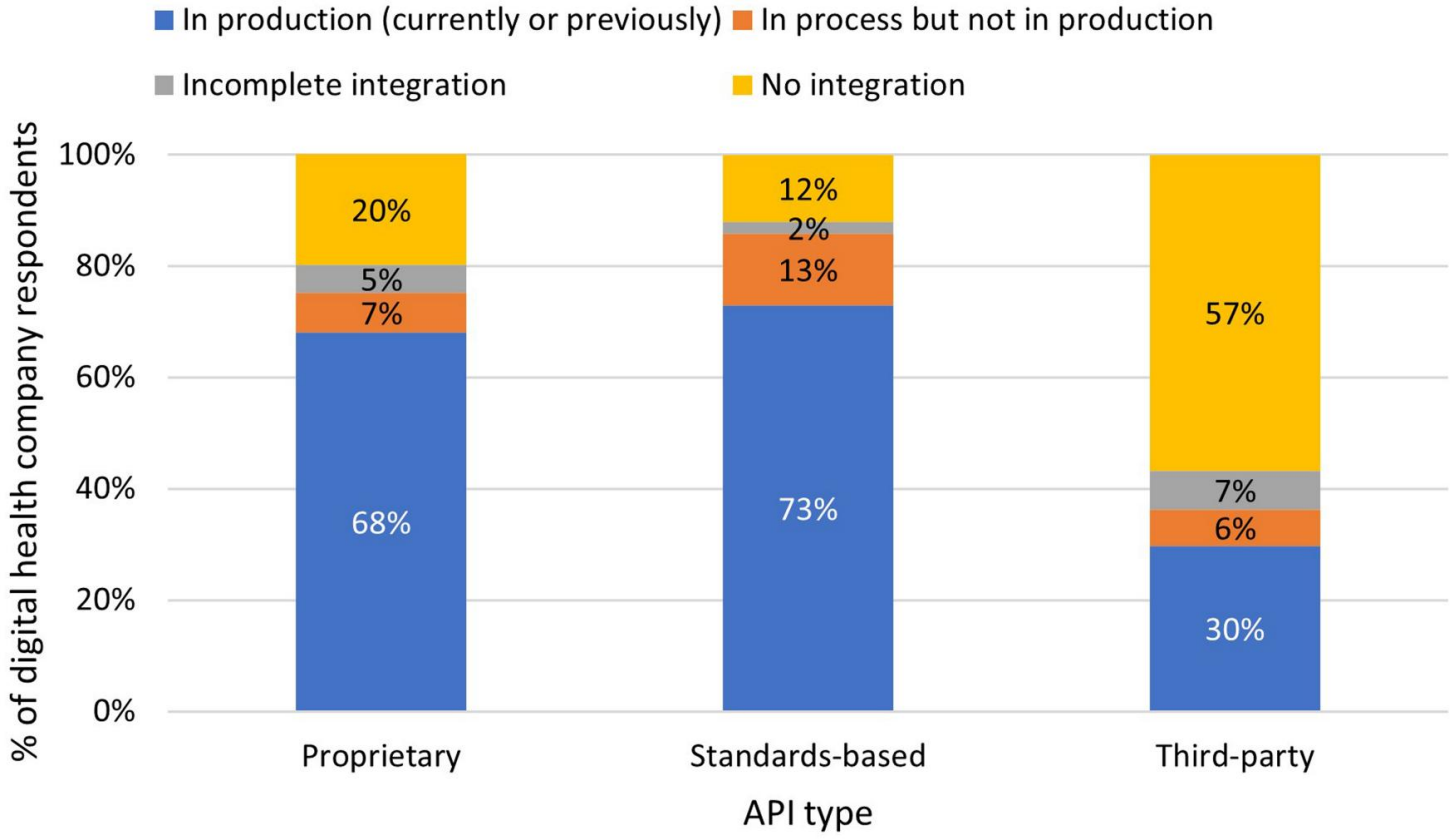
Digital health company status of integrations with EHRs. *N* = 141.

Respondents reported using standards-based APIs to integrate their technologies with EHRs at high levels. Overall, 73% of companies reported current or previous use of a standards-based API in production, and another 13% reported having a standards-based API integration in process (Figure 1). The second most frequently reported method for integration with EHRs was proprietary APIs, which 68% of companies reported as having currently or previously in production. About 30% of respondents indicated currently using or having previously used a third-party integration service in production. It was more common for companies to integrate their solutions using the EHR APIs directly than using a third-party integrator.

**Figure 2.**
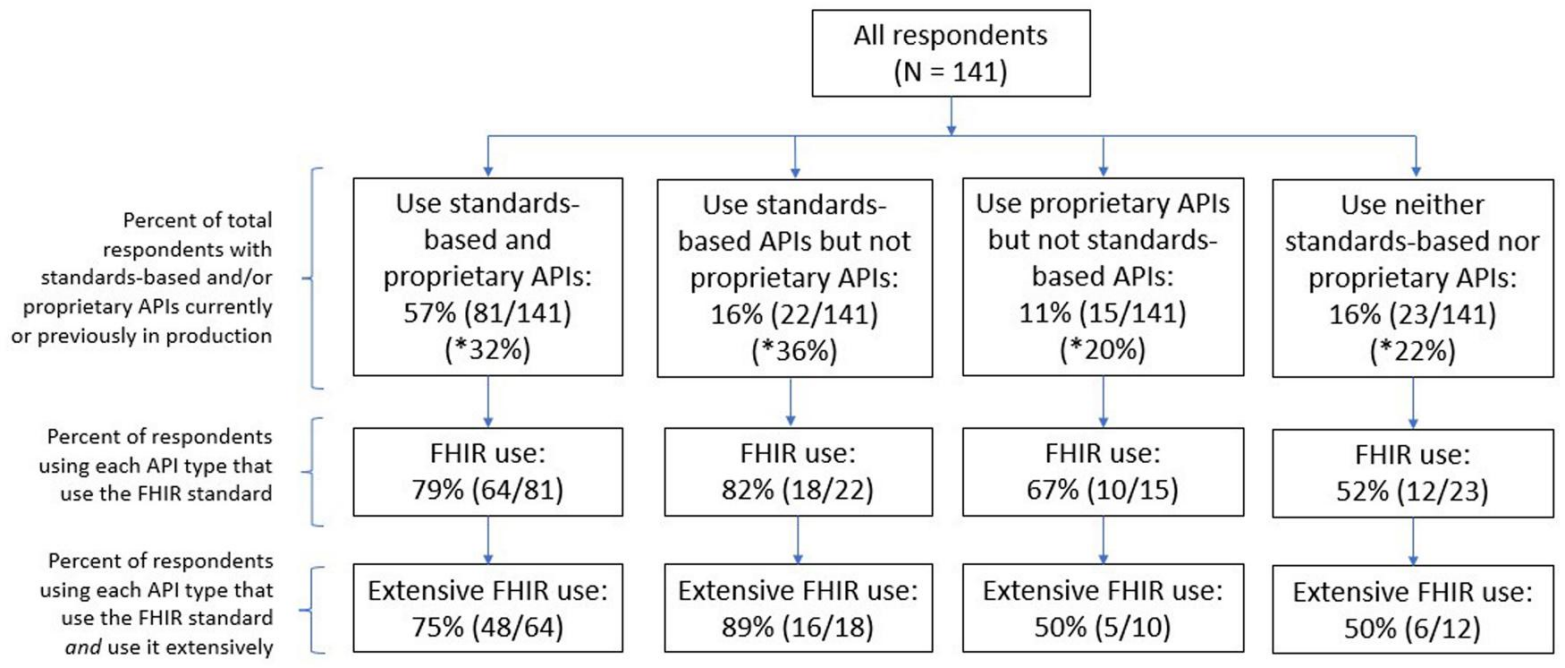
Digital health company use of APIs and the FHIR standard. *N* = 141.

A majority of respondents (57%) indicated using both standards-based and proprietary APIs to integrate with an EHR (Figure 2). Overall, 85% of companies reported supporting the FHIR standard as part of their application, with 61% using the standard extensively. Reported use of the FHIR standard was much higher among companies that used a standards-based EHR API (either alone or alongside a proprietary EHR API) compared to those that did not. 82% of companies using standards-based EHR APIs only and 79% of companies using standards-based EHR APIs alongside proprietary APIs reported use of FHIR in their products, with 89% and 75% of those companies using FHIR, respectively, reporting extensive use of the standard. Conversely, fewer companies that did not use standards-based EHR APIs used the FHIR standard. About 67% of companies only using proprietary APIs to integrate with an EHR and 52% of companies using neither API type, reported use of FHIR, with 50% of those companies using FHIR reporting extensive use of the standard.

**Figure 3.**
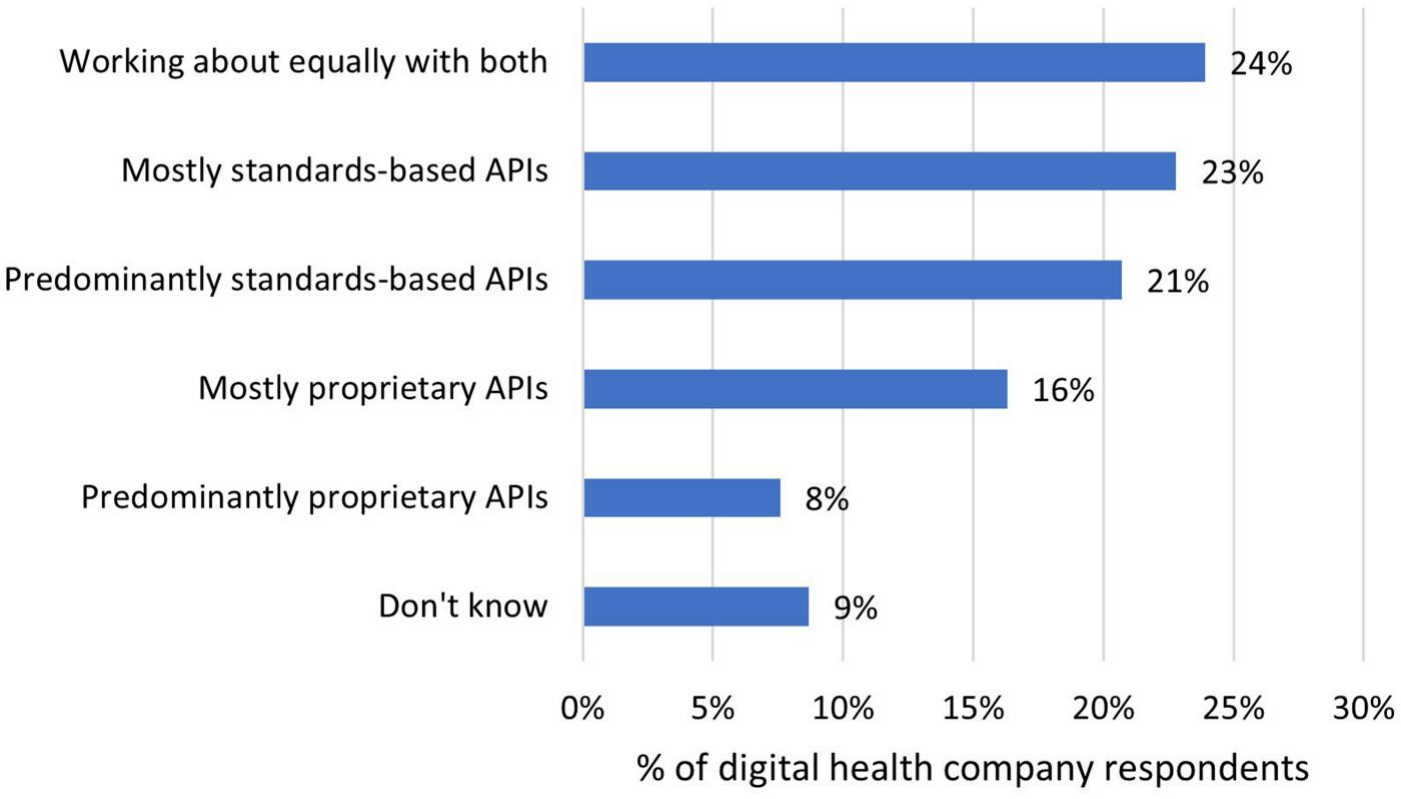
The extent to which digital health companies report currently working with proprietary versus standards-based EHR APIs. *N* = 141.

We found that 24% of companies worked about equally with both standards-based and proprietary APIs and 44% mostly or predominantly used standards-based APIs (Figure 3).

### EHR vendors

**Figure 4.**
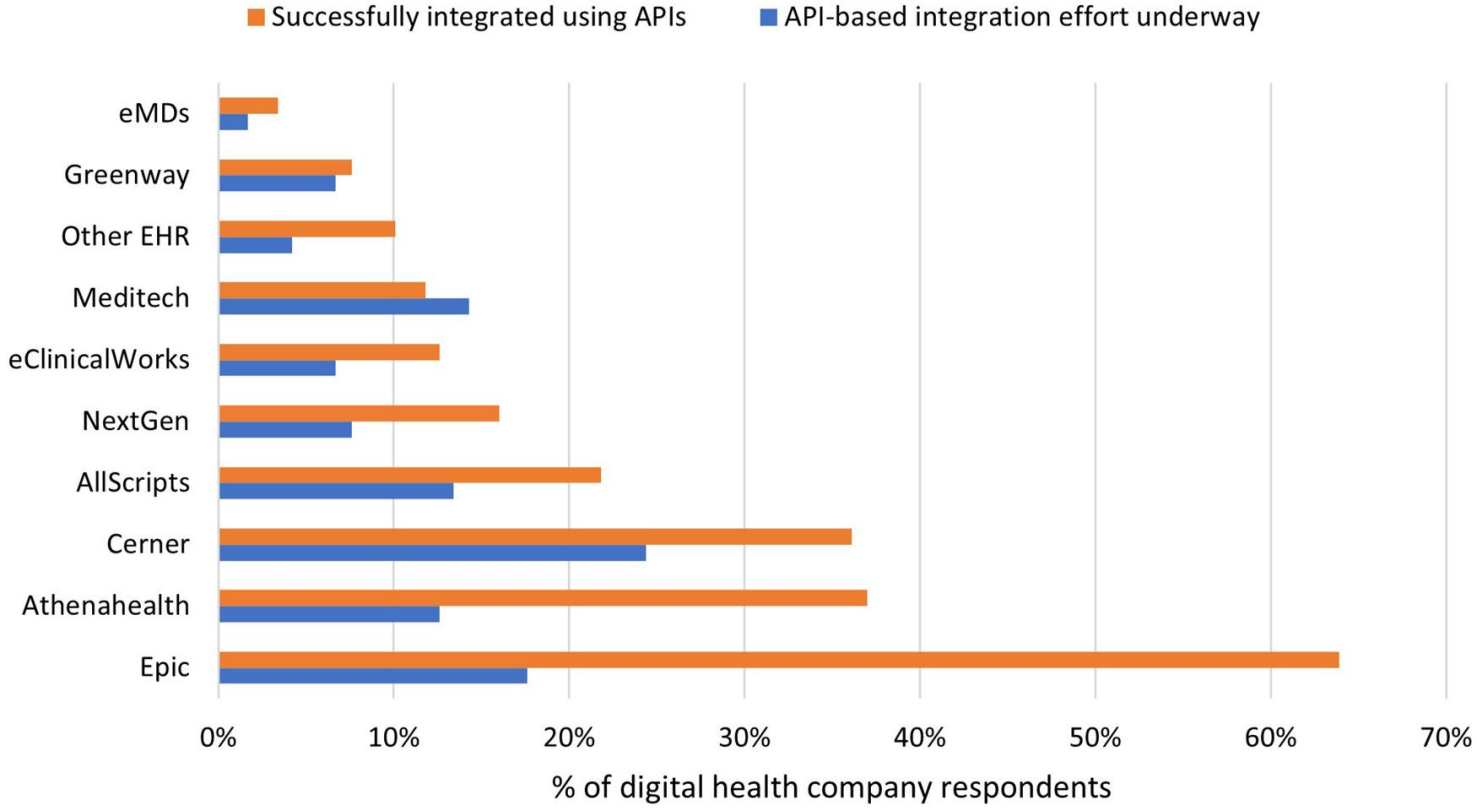
Status of integrations using varying EHR APIs. *N* = 141.

Companies reported successful integrations most frequently with market leading EHRs, including Epic (64%), athenahealth (37%), and Cerner (36%). An additional 18% (Epic), 13% (athenahealth), and 24% (Cerner) of companies reported that API-based integration efforts were underway (Figure 4).

**Figure 5.**
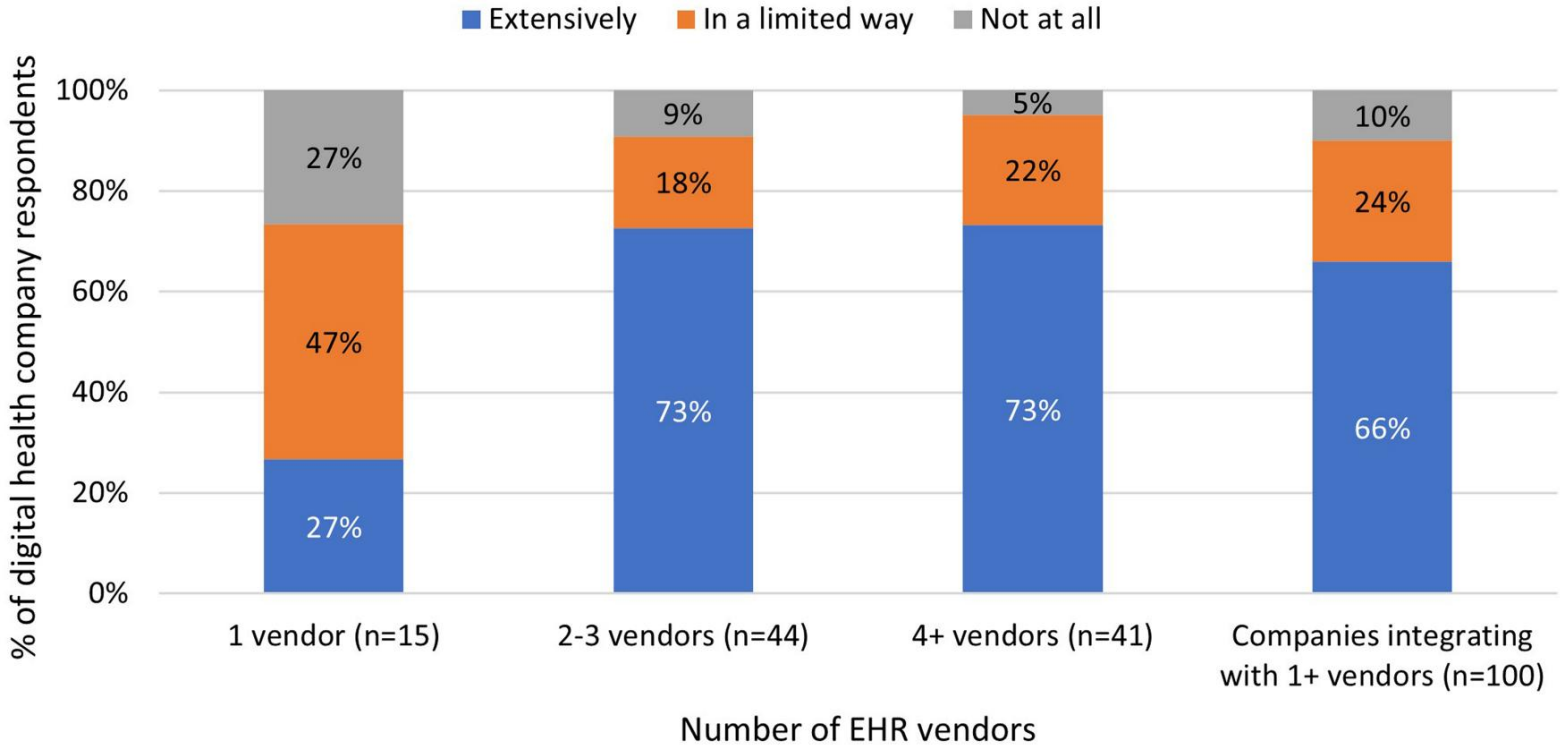
Digital health company respondent use of the FHIR standard, stratified by the number of EHR vendors with which their apps are integrated. *N* = 141.

About 92% of companies had integrations underway with at least 1 EHR and 78% had integrations underway with 2 or more EHRs. Those companies that worked with more than 1 EHR vendor more frequently reported extensive use of the FHIR standard (Figure 5). Specifically, among companies that worked with more than 1 EHR vendor, 73% reported extensive use of FHIR, compared to 27% of companies working with just 1 EHR vendor. About 47% of companies with integrations with just 1 EHR vendor reported using FHIR in a limited way, and 27% reported no use of the FHIR standard. The percent of companies that reported no use of the FHIR standard was just 9% for companies with integrations with 2-3 EHR vendors and 5% for companies with integrations with 4 or more vendors.

### Enablers and barriers

Several dimensions were identified by most respondents as critical for a company’s ability to work successfully with APIs (Table 2). Technical performance (61%), breadth of data elements (60%), cost (56%), and quality documentation (51%) were reported most frequently as dimensions that were critical “to a great extent” for successful work with APIs, followed by EHR vendor support (50%), and effort to implement (45%).

**Table 2.** Percent of digital health company respondents that indicated dimensions were “moderately critical” and “critical to a great extent” for a company’s ability to work successfully with EHR APIs (*N* = 141).

Critical dimension	Moderately critical (%)	Critical to a great extent (%)
Technical performance	30	61
Breadth of data elements	35	60
Cost	34	56
Quality documentation	44	51
EHR vendor support	36	50
Effort to implement	47	45

About 28% of companies rated standards-based APIs as very good based on the critical dimensions for a company to be able to work successfully with an API; this was a larger percent than proprietary APIs (25%), but a lesser percent compared to API-based third-party integration (40%).

**Figure 6.**
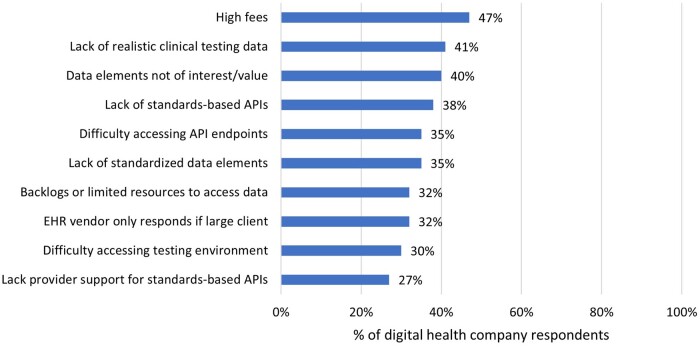
Top 10 “substantial” barriers to integrate with EHRs via APIs. *N* = 141.

Barriers pose challenges to digital health company use of EHR APIs. Among companies that reported using APIs, 47% reported high fees associated with accessing an EHR API as a substantial barrier (Figure 6). The next most common challenges included a lack of realistic clinical testing data (41%), access to data elements of interest or value through APIs (40%), availability of standards-based APIs from the EHR vendor (38%), and standardized data elements (35%).

Efforts to establish and maintain proprietary and standards-based APIs differed substantially. Companies reported that standards-based APIs required on average less burden than proprietary APIs to establish and maintain, with 52% and 21% of companies reporting that substantial effort is required for establishment and maintenance of proprietary APIs, and just 40% and 13% reporting substantial effort required for the establishment and maintenance of standards-based APIs.

Digital health company respondents provided open-ended responses regarding high-priority clinical data types for future federally regulated availability via EHR APIs, as well as future opportunities for policy efforts to promote or enforce access to data. This is summarized in Table 3.

**Table 3.** Five most requested improvements to high-priority clinical data types and future policy opportunities (*N* = 141).

High-priority clinical data types for future federally regulated availability via commercial EHR APIs	Future directions for policy efforts in promoting or enforcing access to data via commercial EHR vendor or payer IT system APIs or other means
Social determinants of health/patient demographics	Enforcement on and incentivization of EHR vendors to follow API standards
Genomics data/testing results	Cost controls on integration, enabling more API access for third party app developers
Prescriptions and administered medications	Conformance testing and other validation tools for APIs
Clinical notes	Expanded data element availability and standards for bulk data transfer/exchange
Claims data	Encourage EHR vendors to support write access in APIs

In brief, respondents indicated interest in federally regulated availability (through EHR APIs) of social determinants of health (SDoH) and demographic data, genomic testing results, prescription and administered medications lists, clinical notes, and claims data. In addition to expanded data element availability, companies frequently highlighted the need for cost controls on EHR integration, as well as enforcement and incentivization of EHR vendor adherence to API standards.

### Non-response bias analysis

Given the survey’s relatively low response rate (20%), we assessed non-response bias and found a few statistically significant differences between respondents and non-respondents. However, the observed, small-magnitude differences are unlikely to bias the representativeness of our results (Appendix SA2).

## Discussion

This study sought to capture current digital health company experiences integrating with EHRs, now that new federally regulated standards-based API policies are in place and being implemented by EHR vendors. Our analysis focused on 3 domains: the use of standards-based and proprietary EHR APIs, integrations across EHR vendors, and enablers and barriers to integrate with EHR APIs. Our results reveal that the majority of respondents use standards-based APIs to integrate with EHRs and support use of the HL7 FHIR standard in their products, likely facilitating their use of standards-based APIs. Although nearly the same number of companies reported use of proprietary EHR APIs, more companies reported predominantly or mostly using standards-based versus proprietary APIs, signaling that both API types were needed to successfully integrate, but that standards-based APIs were more integral. Taken together, this suggests that the field is making important progress in moving toward use of APIs that streamline data exchange through a common language but that a notable portion of digital health companies rely to some extent on non-standards-based APIs.

Substantial barriers such as high fees, lack of realistic clinical testing data, and lack of data elements of interest or value, indicate that progress has not been without associated friction. This is further supported by the significant difference we found in companies’ reported efforts to establish and maintain EHR API integrations—where efforts to establish were more than twice as burdensome. Companies’ recommendations for improving upon the current state of integration included that federal policy should promote more access through cost controls, testing and validation, and an expanded set of data elements available through APIs, which directly address these barriers. Further private sector support and federal policy are needed to ensure APIs are available to reduce barriers to entry and nurture competition “without special effort.”

In particular, results signal an opportunity for industry and ONC to consider and gain input on other high value use cases not currently adopted in the United States Core Data for Interoperability standard and standards-based APIs. Government and industry efforts, through pilots, standards accelerators, and standards development work groups, can help further standardize the data elements that can be accessed using standards-based APIs.^11^ ONC also accepts and uses public feedback and complaints on real-world certified health IT use and barriers through the ONC Health IT Feedback portal to inform agency actions.^12^

Reported barriers related to the uneven availability of APIs and access across different EHRs could lead more digital health companies to focus their integration efforts and customer recruitment across a subset of EHR vendors who provide more robust developer support and a wider availability of data elements beyond just the floor set by federal requirements. The percent of companies, however, that integrate with each EHR vendor align with the EHR market share we calculated across office-based sites and acute care hospitals derived from recent public data sources.^13^^,^^14^ Even though the EHR marketplace skews toward a few predominant market leaders, it is important to ensure the market remains competitive and the burgeoning app ecosystem is built across all technologies (not just a few leaders). High rates of FHIR use among respondents, especially among companies working with multiple EHR vendors, suggest that FHIR-based APIs are successful in supporting apps developed with the intention to scale across multiple EHRs.

### Limitations

The sample and respondents may not comprise a representative sample of digital health companies or all companies that are actively integrating and using EHR APIs. Nonetheless, our methodology to base our sample primarily on a list of companies pulled from public app galleries maintained by EHR vendors and other organizations and evolve and modify that list based on technical expert input resulted in a comprehensive list that, to our knowledge, exists nowhere else. We found through our market research no other representative list or sampling frame for this study, so novel methods and expert insights were needed to derive a sample of companies knowledgeable and experienced to answer the survey’s technical questions.

Our study was also limited to primarily commercial users of EHR APIs and did not include perspectives from clinicians, academic medical center researchers, and other EHR data users, who have research and business cases to use the APIs to connect and integrate their technologies and applications to the EHR. Their perspectives are no less important but were determined as out of scope for this study.

## Conclusion

This study used a novel survey and sampling methodology to derive a robust sample of digital health companies to glean novel, national insights into companies’ experiences using EHR APIs and how the industry and federal policy can continue to shape the healthcare technology ecosystem. We found that a high proportion of digital health companies use standards-based APIs to interoperate with EHRs and support standards as part of their product base. The results show that an iterative and inclusive approach that incorporates industry feedback (not just EHRs, but the digital health and app developer community, too) can help push the technical and functional properties of standards-based APIs forward and in step with developer needs. Continuing to improve the resources for digital health companies to find, test, connect, and use these APIs “without special effort” will be crucial to ensure the technology is robust and durable into the future.

## Supplementary Material

ocae006_Supplementary_Data

## Data Availability

The data underlying this article, even deidentified data, cannot be shared publicly with outside groups to preserve the privacy of individual survey responses. We are able to share aggregated results upon request.
