# Supplementary material for: A national survey of digital health company experiences with electronic health record application programming interfaces
Source: J Am Med Inform Assoc. 2024 Jan 27;31(4):866–74. doi: 10.1093/jamia/ocae006 (PMC10990546; doi:10.1093/jamia/ocae006)
Supplement: ocae006_Supplementary_Data [file ocae006_supplementary_data.doc]

**APPENDIX A: Tables**

**Appendix Table A1**: List of members of national expert advisory board and their respective organizations.

| **Name** | **Organization** |
| --- | --- |
| Michelle Allen | Premier Inc |
| Veenu Aulakh | Independent |
| Meryl Bloomrosen | Premier Inc |
| Hans Buitendijk | HIMSS EHRA, Cerner/Oracle |
| Chris Cameron | Epic |
| Christina Campos | Guadalupe County Hospital |
| Patrick Conway | Optum |
| Cait DesRoches | OpenNotes |
| Stephanie Fiore | Elevance Health |
| Will Gordon | Partners HealthCare |
| Ryan Howells | CARIN Alliance, Leavitt Partners |
| Brendan Keeler | Zus Health |
| Omkar Kulkarni | Children's Hospital Los Angeles (CHLA) |
| Khoa Nguyen | Independent |
| Lucia Savage | Omada Health |
| Mark Savage | Savage & Savage LLC, Gravity Project |
| Alya Sulaiman | Epic |
| Omid Toloui | Elevance Health |
| Sheryl Turney | Carelon Digital Platforms, Elevance Health |

**Appendix Table A2:** Non-response analysis

| **Number of apps for each company listed across all data sources** | | | |
| --- | --- | --- | --- |
| *App count* | *Respondents (%)* | *Non-Respondents (%)* | *Sig. Difference* |
| 1 | 94 (77.7) | 401 (83.1) |  |
| 2 | 20 (16.5) | 56 (11.6) |  |
| 3+ | 7 (5.8) | 25 (5.2) |  |
| **Functional categories tagged for each app** | | | |
| *Category* | *Respondents (%)* | *Non-Respondents (%)* | *Sig. Difference* |
| Administrative | 32 (26.4) | 107 (22.2) |  |
| Clinical Use | 69 (57) | 249 (51.7) |  |
| Patient Care | 84 (69.4) | 323 (67) |  |
| Patient Engagement | 46 (38) | 155 (32.2) |  |
| **Number of galleries where company is listed** | | | |
| *Gallery count* | *Respondents (%)* | *Non-Respondents (%)* | *Sig. Difference* |
| 1 | 80 (66.1) | 364 (75.5) | * |
| 2 | 22 (18.2) | 80 (16.6) |  |
| 3+ | 19 (15.7) | 38 (7.9) | ** |
| Notes: * = p<0.10; ** = p<0.05. Of the 713 companies in the final sample, 704 were part of the original sampling frame and nine were added during fielding. Of the 704 original companies, 603 were sourced from public app marketplaces and galleries managed by EHR companies and not-for-profit organizations. Among these 704 companies, only the 603 sourced from these public data had common variables that could be compared to determine non-response bias. Of the 141 respondents, 121 were sourced from these public app galleries. Non-response analysis for these 121 respondents and 482 non-respondents from the 603 companies sourced from the app galleries are included in the table. | | | |

**APPENDIX B: Survey Instrument**

### INTRODUCTION & CONSENT

**National Survey on Progress and Challenges with APIs:
Perspectives from Digital Health Companies**

This national survey is part of an ongoing biennial study of digital health companies. The goal is to help national decision-makers, and those involved in federal regulation of application programming interfaces (APIs), understand the challenges, as well as the progress, in integrating digital health tools with commercial electronic health records (EHRs) and payers. 

**Survey questions are likely best answered by someone with product expertise but may require some technical expertise as well. Feel free to involve any relevant members of your team in responding. Participation is completely voluntary and will contribute to a research study.** **By completing this survey, you are consenting to participate in the study.**Thank you in advance for your time.

**Data Reporting: What Data & Derivative Results Will be Reported**
No individual respondents or responses will ever be identified or reported publicly. All data will be reported at an aggregate level (e.g., across all survey responses). For example, we may report that 50% of digital health companies have attempted an integration with a commercial electronic health record (EHR) vendor.

**Data Access: Who Will Have Access to Individual, Identified Survey Responses**
The UCSF research team that is collecting the data will have access to complete survey responses. In addition, the California Health Care Foundation (CHCF), the Office of the National Coordinator for Health IT (ONC) and ScaleHealth will be given a dataset containing identifiable survey responses. ONC may choose to share all or part of the dataset with ONC contractors only for the purpose of conducting contract work and abiding by the same reporting/disclosure terms as described above. Data access is for research purposes only and will not be used to determine conformance with certification requirements or other regulations.

### SECTION 1: EXPERIENCE WITH EHR INTEGRATIONS

Which best describes your company’s relationship with Protected Health Information (PHI)? *Hover mouse over bolded text for definitions*

Healthcare Provider or Other Covered Entity [Q1_1]

Business Associate of a Covered Entity [Q1_2]

Access PHI through consumers outside Business Associate or Covered Entity Relationship [Q1_3]

Other (please specify) [Q1_4]:       [Q1_4_TEXT]

***The subsequent questions ask about API-based integrations with commercial EHRs.***

***Specifically, we are interested in your experiences using RESTful API approaches.*** *RESTful APIs are a type of web API that uses internet URLs to securely access data from a website or server (i.e., those using HTTP protocols). Common examples of non-RESTful APIs and other approaches to EHR integration include HL7v2, CDA exchange, flat files over SFTP (secure file transfer protocol), and RPA (robotic process automation).*

Has your company attempted any of the following API-based integrations with commercial EHR(s): (this question is referred to as “matrix question” in display logic instructions).

| Integration with commercial EHR(s) via… | **Yes, In Production** (Currently or previously) | **Yes, In Process but Not In Production** | **Yes, but Stopped** (Incomplete) | **No** |
| --- | --- | --- | --- | --- |
| *Proprietary* RESTful Application Programming Interfaces (APIs) [Q2_1] | ☐ | ☐ | ☐ | ☐ |
| *Standards-based* RESTful Application Programming Interfaces (APIs)* [Q2_2] | ☐ | ☐ | ☐ | ☐ |
| An API-based third-party integration engine (e.g., Redox, 1upHealth) [Q2_3] | ☐ | ☐ | ☐ | ☐ |

* This refers to the APIs required under the 21^st^ Century Cures Act, as implemented by CMS and ONC.

Is your company currently integrating with commercial EHRs using non-RESTful APIs or integration approaches that do not rely on APIs? [Q3]

Yes. Please briefly describe approaches:      [Q3_TEXT]

No

Don’t Know

**Display if “Yes” to above question:** To what extent do you agree with the following statement:

*We are using approaches that do not rely on RESTful APIs because current RESTful APIs are not able to meet our business needs.* [Q4]

| **Strongly Agree** | **Somewhat Agree** | **Neutral** | **Somewhat Disagree** | **Strongly Disagree** | **Don’t Know** |
| --- | --- | --- | --- | --- | --- |
| ☐ | ☐ | ☐ | ☐ | ☐ | ☐ |

**From this point forward, the term APIs is used to refer to RESTful APIs.**

**Display if they did not select all No’s in the matrix question.** Did all API-based integrations with commercial EHR vendors occur before 2017? [Q5]

Yes (skip to [end of this section of questions](#_BRANCH_OPTIONS_BASED))

No

Don’t Know

**Display if answer to above question is “No” or “Don’t Know.”** Between January 2017 and the present, when did API integrations with commercial EHR vendors occur? [Q6]

All between January 2017 – December 2019

Some Before/ Some After

All since January 2020

Don’t Know

**Display if any “Yes” response in row 3 of matrix question.** Please list the API-based third-party integration engine(s) that you currently use or have you used in the past:

CURRENTLY USE:       [Q7]

USED IN THE PAST:       [Q8]

**Display if any “Yes” response in row 3 of matrix question.** Please briefly describe your rationale for working with an API-based third-party integration engine(s):

      [Q9]

### BRANCH OPTIONS BASED ON ANSWERS TO MATRIX QUESTIONS:

- [**Any “Yes in Production” or “Yes in Process” Responses**](file:///C:/Users/jadlermilstein/AppData/Local/Microsoft/Windows/INetCache/Content.Outlook/9X9HO5CJ/YES_IN_1#_ANY_)
- [**Combo of “Yes but Stopped” and “No”**](file:///C:/Users/jadlermilstein/AppData/Local/Microsoft/Windows/INetCache/Content.Outlook/9X9HO5CJ/YES_1#_COMBINATION_OF_)
- [**All “No”**](file:///C:/Users/jadlermilstein/AppData/Local/Microsoft/Windows/INetCache/Content.Outlook/9X9HO5CJ/NO#_ALL_)

###

### ANY “YES IN PRODUCTION” OR “YES IN PROCESS” RESPONSES

**Please respond to the following questions based on your product(s) that integrate or are in the process of integrating with commercial EHRs using APIs.**

What are the primary application domains of your product(s) that integrate/will integrate with commercial EHRs using APIs? Select all that apply:

Administrative (e.g., scheduling, billing, check-in) [Q10_1]

Care Delivery, not limited to Treatment (e.g., clinical decision support, care coordination, telehealth, remote patient monitoring, clinical messaging) [Q10_2]

Clinical Research [Q10_3]

Patient Access and Management of Health Record Data [Q10_4]

Population Health (including analytics, measurement, and reporting) [Q10_5]

Public Health [Q10_6]

Other (please specify): [Q10_7]       [Q10_7_TEXT]

Who are the primary buyer(s) and user(s) of your product(s) that integrate with commercial EHRs using APIs? Select all that apply.

|  | **Buyer(s)**  *Those who pay you for your products that integrate with commercial EHRs* | **User(s)**  *Those who use your products that integrate with commercial EHRs* |
| --- | --- | --- |
| Not Applicable (We only integrate for internal business purposes, and don't sell those services to others). | ☐ [Q11_1_1] | [Q11_1_2] |
| Individual Clinician | ☐ [Q11_2_1] | ☐ [Q11_2_2] |
| Patient or Caregiver | ☐ [Q11_3_1] | ☐ [Q11_3_2] |
| Provider Organization | ☐ [Q11_4_1] | ☐ [Q11_4_2] |
| Payer | ☐ [Q11_5_1] | ☐ [Q11_5_2] |
| Public Health Organization | ☐ [Q11_6_1] | ☐ [Q11_6_2] |
| Other Government Agency | ☐ [Q11_7_1] | ☐ [Q11_7_2] |
| Qualified Clinical Data Registry | ☐ [Q11_8_1] | ☐ [Q11_8_2] |
| Health Information Exchange Organization | ☐ [Q11_9_1] | ☐ [Q11_9_2] |
| Digital Health Company | ☐ [Q11_10_1] | ☐ [Q11_10_2] |
| Pharmaceutical Company | ☐ [Q11_11_1] | ☐ [Q11_11_2] |
| Medical Device Company | ☐ [Q11_12_1] | ☐ [Q11_12_2] |
| Commercial Laboratories | ☐ [Q11_13_1] | ☐ [Q11_13_2] |
| Revenue Cycle Management Company | ☐ [Q11_14_1] | ☐ [Q11_14_2] |
| Other (please specify):           [Q11_15_TEXT] | ☐ [Q11_15_1] | ☐ [Q11_15_2] |
| Other (please specify):           [Q11_16_TEXT] | ☐ [Q11_16_1] | ☐ [Q11_16_2] |

**Display if they don’t check “N/A” in prior question:** Do you make any of your API-based product(s) available via the following? Select all that apply.

Commercial EHR vendor(s) app store (e.g., Epic App Orchard, Cerner App Gallery) [Q12_1]

App stores marketed for smartphones (e.g., Apple, Google Play) [Q12_2]

Other, non-commercial app store (e.g., SMART, CARIN, BlueButton, VA) [Q12_3]

Other (please specify): [Q12_4]       [Q12_14_TEXT]

How many API-based apps do you make that integrate with commercial EHRs? [Q13]

1

2-5

6+

In what stage of development is your company? [Q14]

Incubation (Pre-Seed, Seed)

Early stage (Series A & B)

Development, Growth (Series C or later)

Public or Acquired by a Public Company

Other (please specify):       [Q14_TEXT]

Approximately how many full-time equivalent (FTE) staff work on your product(s) that integrate with commercial EHRs? [Q15]

1-10

11-50

51 – 100

101 - 250

250- 500

More than 500

In what regions do you have existing clients of your product(s) that integrate with commercial EHRs? Select all that apply.

US – Northeast [Q16_1]  Mexico [Q16_6]

US – Midwest [Q16_2]  Europe [Q16_7]

US – South [Q16_3]  Asia/Pacific [Q16_8]

US – West [Q16_4]  Africa/Middle East [Q16_9]

Canada [Q16_5]  Central/South America [Q16_10]

With how many *client EHR instances** have you:

|  | 1-5 | 6-9 | 10-99 | 100+ |
| --- | --- | --- | --- | --- |
| **Attempted API-based integration but stopped** [Q17_1] | ☐ | ☐ | ☐ | ☐ |
| **API-based Integration effort underway** [Q17_2] | ☐ | ☐ | ☐ | ☐ |
| **Successfully integrated using APIs** [Q17_3] | ☐ | ☐ | ☐ | ☐ |

* *For example, if you have a single client with 5 different EHR instances with which you integrated/attempted to integrate,* ***count as 5***

With which specific commercial EHR vendors have you:

|  | **Never attempted an API-based integration** | **Attempted API-based integration but stopped** | **API-based Integration effort underway** | **Successfully integrated using APIs** |
| --- | --- | --- | --- | --- |
| Allscripts [Q18_1] | ☐ | ☐ | ☐ | ☐ |
| athenahealth [Q18_2] | ☐ | ☐ | ☐ | ☐ |
| Cerner [Q18_3] | ☐ | ☐ | ☐ | ☐ |
| eClinicalWorks [Q18_4] | ☐ | ☐ | ☐ | ☐ |
| eMDs [Q18_5] | ☐ | ☐ | ☐ | ☐ |
| Epic [Q18_6] | ☐ | ☐ | ☐ | ☐ |
| Greenway [Q18_7] | ☐ | ☐ | ☐ | ☐ |
| Meditech [Q18_8] | ☐ | ☐ | ☐ | ☐ |
| NextGen [Q18_9] | ☐ | ☐ | ☐ | ☐ |
| Other (please specify) [Q18_10] :  [Q18_10_TEXT] | ☐ | ☐ | ☐ | ☐ |

**If “Yes in Production” or “Yes in Process” to both standards-based and proprietary APIs in matrix question ask:** To what extent are you currently working with commercial EHR vendors’ proprietary APIs versus standards-based APIs? [Q19]

| **Predominantly proprietary APIs** | **Mostly proprietary APIs** | **Working about equally with both** | **Mostly standards-based APIs** | **Predominantly standards-based APIs** | **Don’t Know** |
| --- | --- | --- | --- | --- | --- |
| ☐ | ☐ | ☐ | ☐ | ☐ | ☐ |

**Based on responses to matrix question, filter which row(s) are shown**: Please rate the overall level of effort/resources to **establish** integrations with commercial EHR vendors?

|  | **INITIAL IMPLEMENTATION** | | | **Don’t Know** |
| --- | --- | --- | --- | --- |
| Integration with commercial EHR(s) via… | **Minimal** | **Moderate** | **Substantial** |  |
| *proprietary* RESTful Application Programming Interfaces (APIs) [Q20_1] | ☐ | ☐ | ☐ | ☐ |
| *standards-based* RESTful Application Programming Interfaces (APIs)† [Q20_2] | ☐ | ☐ | ☐ | ☐ |
| an API-based third-party integration engine (e.g., Redox, 1upHealth) [Q20_3] | ☐ | ☐ | ☐ | ☐ |

**Based on responses to matrix question, filter which row(s) are shown**: Please rate the overall level of effort/resources to **maintain** integrations with commercial EHR vendors?

|  | **ONGOING USE/MAINTENANCE** | | | **Don’t Know** |
| --- | --- | --- | --- | --- |
| Integration with commercial EHR(s) via… | **Minimal** | **Moderate** | **Substantial** |  |
| *proprietary* RESTful Application Programming Interfaces (APIs) [Q21_1] | ☐ | ☐ | ☐ | ☐ |
| *standards-based* RESTful Application Programming Interfaces (APIs)† [Q21_2] | ☐ | ☐ | ☐ | ☐ |
| an API-based third-party integration engine (e.g., Redox, 1upHealth) [Q21_3] | ☐ | ☐ | ☐ | ☐ |

To what extent is each dimension critical to your ability to successfully work with the API:

| **Critical Dimensions** | **To a great extent** | **Moderately** | **Minimally** | **Not at all** |
| --- | --- | --- | --- | --- |
| Technical performance (e.g., response time) [Q22_1] | ☐ | ☐ | ☐ | ☐ |
| Cost (e.g., fees) [Q22_2] | ☐ | ☐ | ☐ | ☐ |
| Quality of API documentation [Q22_3] | ☐ | ☐ | ☐ | ☐ |
| EHR vendor support during development and implementation [Q22_4] | ☐ | ☐ | ☐ | ☐ |
| Breadth of data elements available [Q22_5] | ☐ | ☐ | ☐ | ☐ |
| Amount of effort required to implement [Q22_6] | ☐ | ☐ | ☐ | ☐ |
| Other (please specify): [Q22_7]      [Q22_7_TEXT] | ☐ | ☐ | ☐ | ☐ |

**Based on responses to matrix question, filter which row(s) are shown**: Based on the critical dimensions you rated "to a great extent" or "moderately" above, how would you rate the APIs you gained access to in the course of the integration process?

| Integration with commercial EHR(s) via… | **Very Good** | **Fair** | **Poor** | **N/A** | **Don’t Know** |
| --- | --- | --- | --- | --- | --- |
| *proprietary* RESTful Application Programming Interfaces (APIs) [Q23_1] | ☐ | ☐ | ☐ | ☐ | ☐ |
| *standards-based* RESTful Application Programming Interfaces (APIs)† [Q23_2] | ☐ | ☐ | ☐ | ☐ | ☐ |
| an API-based third-party integration engine (e.g., Redox, 1upHealth) [Q23_3] | ☐ | ☐ | ☐ | ☐ | ☐ |

**Based on responses to matrix question, filter which row(s) are shown**: Based on the critical dimensions you rated "to a great extent" or "moderately" above, how would you rate the change over the past year in the APIs you gained access to in the course of the integration process?

| Integration with commercial EHR(s) via… | **Much better (than 12 mos ago)** | **Somewhat better** | **About the same** | **Somewhat worse** | **Much worse** | **N/A** | **Don’t Know** |
| --- | --- | --- | --- | --- | --- | --- | --- |
| *proprietary* RESTful Application Programming Interfaces (APIs) [Q24_1] | ☐ | ☐ | ☐ | ☐ | ☐ | ☐ | ☐ |
| *standards-based* RESTful Application Programming Interfaces (APIs)† [Q24_2] | ☐ | ☐ | ☐ | ☐ | ☐ | ☐ | ☐ |
| an API-based third-party integration engine (e.g., Redox, 1upHealth) [Q24_3] | ☐ | ☐ | ☐ | ☐ | ☐ | ☐ | ☐ |

Based on the critical dimensions you rated "to a great extent" or "moderately" above, which commercial EHR vendor(s) with whom you have tried to integrate do you consider the strongest and which need the most improvement?

STRONGEST VENDORS (please list up to 3):       [Q25_1]

VENDORS THAT NEED MOST IMPROVEMENT (please list up to 3):        [Q25_2]

Among the commercial EHR vendor(s) that you have worked with, which would you say have the strongest partner programs (e.g., sandboxes, documentation, access to support/help) and which provide the least support for API-based integration?

VENDORS WITH STRONGEST API-INTEGRATION PARTNER PROGRAMS (please list up to 3):

      [Q26_1]

VENDORS THAT PROVIDE LEAST SUPPORT FOR API INTEGRATION (please list up to 3):

      [Q26_2]

Please indicate the current level of use for your API-based commercial EHR integrations:

|  | **CURRENT USE** | | |
| --- | --- | --- | --- |
| **Functions** | **Use extensively** | **Use in a limited way** | **Do not use** |
| Create (“write”) [Q27_1] | ☐ | ☐ | ☐ |
| Read [Q27_2] | ☐ | ☐ | ☐ |
| Update [Q27_3] | ☐ | ☐ | ☐ |
| Delete [Q27_4] | ☐ | ☐ | ☐ |
| **Clinical Data Types*** |  |  |  |
| Allergies and Intolerances [Q27_5] | ☐ | ☐ | ☐ |
| Clinical Notes [Q27_6] | ☐ | ☐ | ☐ |
| Clinical/Laboratory Tests & Results [Q27_7] | ☐ | ☐ | ☐ |
| Conditions/Diagnoses [Q27_8] | ☐ | ☐ | ☐ |
| Immunizations [Q27_9] | ☐ | ☐ | ☐ |
| Medications [Q27_10] | ☐ | ☐ | ☐ |
| Orders [Q27_11] | ☐ | ☐ | ☐ |
| Patient Demographics [Q27_12] | ☐ | ☐ | ☐ |
| Patient Reported Outcome Measures [Q27_13] | ☐ | ☐ | ☐ |
| Vitals Signs [Q27_14] | ☐ | ☐ | ☐ |

* List of clinical data types is intentionally not exhaustive

Please indicate the value of future federally regulated APIs for commercial EHRs that enable:

|  | **FUTURE VALUE** | | |
| --- | --- | --- | --- |
| **Functions** | **High value** | **Moderate value** | **Limited/No value** |
| Create (“write”) [Q28_1] | ☐ | ☐ | ☐ |
| Update [Q28_2] | ☐ | ☐ | ☐ |
| Delete [Q28_3] | ☐ | ☐ | ☐ |

Please list up to five types of clinical data beyond those included in USCDI ([USCDI data element list](https://www.healthit.gov/isa/sites/isa/files/2021-07/USCDI-Version-2-July-2021-Final.pdf)) that you consider high-priority for future federally-regulated availability via commercial EHR APIs:

      [Q29]

Continue to [section 2](#_SECTION_2:_BARRIERS)

### COMBINATION OF “YES BUT STOPPED” AND “NO”

**For any “yes but stopped” in matrix question – filter which question below are shown:**

Please indicate reason(s) why you stopped integration with **proprietary** RESTful Application Programming Interfaces (APIs).

☐ Integration did not add sufficient value to our product(s) or service(s) (e.g., data were not helpful, data did not enhance our product or service) [Q30_1]

☐ Integration required too much engineering effort or other human capital costs [Q30_2]

☐ Integration fees were too costly [Q30_3]

☐ Technical challenges [Q30_4]

☐ Other: [Q30_5] please list  [Q30_5_TEXT]

Please indicate reason(s) why you stopped integration with federally-regulated, **standards-based** RESTful Application Programming Interfaces (APIs). Select all that apply.

☐ Integration did not add sufficient value to our product(s) or service(s) (e.g., data were not helpful, data did not enhance our product or service) [Q31_1]

☐ Integration required too much engineering effort or other human capital costs [Q31_2]

☐ Integration fees were too costly [Q31_3]

☐ Technical challenges [Q31_4]

☐ Other: [Q31_5] please list   [Q31_5_TEXT]

Please indicate reason(s) why you stopped integration with an API-based **third-party integration engine** (e.g., Redox, 1 upHealth). Select all that apply.

☐ Integration did not add sufficient value to our product(s) or service(s) (e.g., data were not helpful, data did not enhance our product or service) [Q32_1]

☐ Integration required too much engineering effort or other human capital costs [Q32_2]

☐ Integration fees were too costly [Q32_3]

☐ Technical challenges [Q32_4]

☐ Other: [Q32_5] please list [Q32_5_TEXT]

Continue to [section 2](#_SECTION_2:_BARRIERS)

### ALL “NO”

**If all “no” in Q2:** Please indicate reason(s) why you haven't integrated with commercial EHR(s) using APIs:

Integration did not add sufficient value to our product(s) or service(s) (e.g., data were not helpful, data did not enhance my product or service) [Q33_1]

Integration required too much engineering effort or other human capital costs [Q33_2]

Integration fees were too costly [Q33_3]

Technical challenges [Q33_4]

Other: [Q33_5] please list       [Q33_5_TEXT]

Continue to section 2 ([specific to “Nos”)](#_SECTION_2:_BARRIERS_1)

### SECTION 2: BARRIERS TO COMMERICAL EHR INTEGRATIONS

To what extent does each of the following pose barriers to your product(s) that integrate with commercial EHRs using APIs.

|  | **Substantial barrier** | | **Moderate barrier** | | **Minor/not a barrier** | | **N/A or Don’t know** | | |
| --- | --- | --- | --- | --- | --- | --- | --- | --- | --- |
| TECHNICAL PERFORMANCE |  | |  | |  | |  | | |
| Length of access/refresh token [Q34_1] | ☐ | | ☐ | | ☐ | | ☐ | | |
| Lack of standardized data elements [Q34_2] | ☐ | | ☐ | | ☐ | | ☐ | | |
| High volume of API calls needed to access data [Q34_3] | ☐ | | ☐ | | ☐ | | ☐ | | |
| Difficulty with identity matching or management [Q34_4] | ☐ | | ☐ | | ☐ | | ☐ | | |
| DATA QUALITY AND AVAILABILITY | |  | |  | |  | |  |  |
| API(s) do not provide access to data elements of interest/value [Q34_5] | | ☐ | | ☐ | | ☐ | | ☐ |  |
| IMPLEMENTATION | |  | |  | |  | |  |  |
| Lack of provider support for standards-based APIs [Q34_6] | | ☐ | | ☐ | | ☐ | | ☐ |  |
| Lack of availability of standards-based APIs **from EHR vendor** [Q34_7] | | ☐ | | ☐ | | ☐ | | ☐ |  |
| Difficulty accessing **provider organization** API endpoints [Q34_8] | | ☐ | | ☐ | | ☐ | | ☐ |  |
| TECHNICAL SUPPORT | |  | |  | |  | |  |  |
| Difficulty accessing **EHR vendor** testing environment [Q34_9] | | ☐ | | ☐ | | ☐ | | ☐ |  |
| Difficulty accessing **EHR vendor** technical API documentation [Q34_10] | | ☐ | | ☐ | | ☐ | | ☐ |  |
| Difficulty accessing **provider organization** testing environment [Q34_11] | | ☐ | | ☐ | | ☐ | | ☐ |  |
| Lack of ability to integrate and test with security framework [Q34_12] | | ☐ | | ☐ | | ☐ | | ☐ |  |
| Lack of realistic clinical testing data in testing or development environments [Q34_13] | | ☐ | | ☐ | | ☐ | | ☐ |  |
| BUSINESS PRACTICES & FEES | |  | |  | |  | |  |  |
| **EHR vendor** denied our access to their API due to concerns about usability or security [Q34_14] | | ☐ | | ☐ | | ☐ | | ☐ |  |
| High fee(s) associated with accessing vendor API or other non-API interface. [Q34_15] | | ☐ | | ☐ | | ☐ | | ☐ |  |
| Contracting terms with **EHR vendor** (e.g. that inhibited our ability to keep intellectual property) [Q34_16] | | ☐ | | ☐ | | ☐ | | ☐ |  |
| **Provider organizations** require HITRUST, SOC2 or other security certifications [Q34_17] | | ☐ | | ☐ | | ☐ | | ☐ |  |
| Backlogs or limited resources at client sites to gain data access, including barriers to getting a Business Associate Agreement (BAA) [Q34_18] | | ☐ | | ☐ | | ☐ | | ☐ |  |
| **EHR vendor** will only respond if asked by large provider client [Q34_19] | | ☐ | | ☐ | | ☐ | | ☐ |  |

Please share any other thoughts or comments on barriers encountered or the general process of integrating with commercial EHRs using APIs. [Q35]

Continue to [Payer Integration Questions](#_PAYER_INTEGRATIONS)

### SECTION 2: BARRIERS (SPECIFIC TO NO’S)

If relevant, to what extent did each of the following barriers contribute to your decision to not integrate your product(s) with commercial EHRs using APIs.

|  | **Substantial barrier** | | **Moderate barrier** | | **Minor/not a barrier** | | **N/A or Don’t know** | | |
| --- | --- | --- | --- | --- | --- | --- | --- | --- | --- |
| TECHNICAL PERFORMANCE |  | |  | |  | |  | | |
| Length of access/refresh token [Q36_1] | ☐ | | ☐ | | ☐ | | ☐ | | |
| Lack of standardized data elements [Q36_2] | ☐ | | ☐ | | ☐ | | ☐ | | |
| High volume of API calls needed to access data [Q36_3] | ☐ | | ☐ | | ☐ | | ☐ | | |
| Difficulty with identity matching or management [Q36_4] | ☐ | | ☐ | | ☐ | | ☐ | | |
| DATA QUALITY AND AVAILABILITY | |  | |  | |  | |  |  |
| API(s) did not provide access to data elements of interest/value [Q36_5] | | ☐ | | ☐ | | ☐ | | ☐ |  |
| IMPLEMENTATION | |  | |  | |  | |  |  |
| Lack of provider support for standards-based APIs [Q36_6] | | ☐ | | ☐ | | ☐ | | ☐ |  |
| Lack of availability of standards-based APIs **from EHR vendor** [Q36_7] | | ☐ | | ☐ | | ☐ | | ☐ |  |
| Difficulty accessing **provider organization** API endpoints [Q36_8] | | ☐ | | ☐ | | ☐ | | ☐ |  |
| TECHNICAL SUPPORT | |  | |  | |  | |  |  |
| Difficulty accessing **EHR vendor** testing environment [Q36_9] | | ☐ | | ☐ | | ☐ | | ☐ |  |
| Difficulty accessing **EHR vendor** technical API documentation [Q36_10] | | ☐ | | ☐ | | ☐ | | ☐ |  |
| Difficulty accessing **provider organization** testing environment [Q36_11] | | ☐ | | ☐ | | ☐ | | ☐ |  |
| Lack of ability to integrate and test with security framework [Q36_12] | | ☐ | | ☐ | | ☐ | | ☐ |  |
| Lack of realistic clinical testing data in testing or development environments [Q36_13] | | ☐ | | ☐ | | ☐ | | ☐ |  |
| BUSINESS PRACTICES & FEES | |  | |  | |  | |  |  |
| **EHR vendor** denied our access to their API due to concerns about usability or security [Q36_14] | | ☐ | | ☐ | | ☐ | | ☐ |  |
| High fee(s) associated with accessing vendor API or other non-API interface. [Q36_15] | | ☐ | | ☐ | | ☐ | | ☐ |  |
| Contracting terms with **EHR vendor** (e.g. that inhibited our ability to keep intellectual property) [Q36_16] | | ☐ | | ☐ | | ☐ | | ☐ |  |
| **Provider organizations** required HITRUST, SOC2 or other security certifications [Q36_17] | | ☐ | | ☐ | | ☐ | | ☐ |  |
| Backlogs or limited resources at client sites to gain data access, including barriers to getting a Business Associate Agreement (BAA) [Q36_18] | | ☐ | | ☐ | | ☐ | | ☐ |  |
| **EHR vendor** would only respond if asked by large provider client [Q36_19] | | ☐ | | ☐ | | ☐ | | ☐ |  |

Please share any other thoughts or comments on barriers encountered or the general process of integrating with commercial EHRs using APIs. [Q37]

Continue to [Payer Integration Questions](#_PAYER_INTEGRATIONS)

### PAYER INTEGRATIONS

Has your company attempted any of the following API-based integrations with payer(s) IT systems (i.e., accessing data held by payers in their information systems): (this question is referred to as payer matrix question in display logic instructions)

| Integration with payer(s) via… | **Yes, In Production** (Currently or previously) | **Yes, In Process but Not In Production** | **Yes, but Stopped Incomplete** | **No** |
| --- | --- | --- | --- | --- |
| *proprietary* RESTful Application Programming Interfaces (APIs) [Q38_1] | ☐ | ☐ | ☐ | ☐ |
| *standards-based* RESTful Application Programming Interfaces (APIs) [Q38_2] | ☐ | ☐ | ☐ | ☐ |
| a third-party integration engine [Q38_3] | ☐ | ☐ | ☐ | ☐ |

Is your company currently integrating with payer(s) IT systems using approaches that do not rely on APIs? [Q39]

Yes. Please briefly describe approaches:       [Q39_TEXT]

No

Don’t Know

**Display if “Yes” to above question:** To what extent do you agree with the following statement:

*We are using approaches that do not rely on APIs because current APIs are not able to meet our business needs.* [Q40]

| **Strongly Agree** | **Somewhat Agree** | **Neutral** | **Somewhat Disagree** | **Strongly Disagree** | **Don’t Know** |
| --- | --- | --- | --- | --- | --- |
| ☐ | ☐ | ☐ | ☐ | ☐ | ☐ |

**Display if “Yes in Production” or “Yes in Process” in payer matrix question.** With which types of payers have you successfully integrated using API-based approaches? [Q41]

**Display if “Yes in Production” or “Yes in Process” in payer matrix question.** Among the payer IT systems(s) that you have worked with, which would you say have the strongest partner programs (e.g., sandboxes, documentation, access to support/help) and which provide the least support for API-based integration?

THOSE WITH STRONGEST API-INTEGRATION PARTNER PROGRAMS (please list up to 3):

      [Q42_1]

THOSE THAT PROVIDE LEAST SUPPORT FOR API INTEGRATION (please list up to 3):

      [Q42_2]

Continue to [section 3](#_SECTION_3:_POLICY)

SECTION 3: POLICY EFFORTS

Fast Healthcare Interoperability Resources (FHIR) is a standard describing data formats and elements. Do you use FHIR in your product(s)? [Q43]

Extensively

In a limited way

Not at all

Don’t know

**Display if “Extensively” or “In a Limited Way” in above question.** If you have developed FHIR-based products, do your products routinely access commercial EHR FHIR servers using SMART on FHIR authorization(s)? [Q44]

Yes

No

Don’t know

To what extent are you investing in developing products around:

|  | **To a great extent** | **Moderately** | **Minimally** | **Not at all** | **Don’t know** |
| --- | --- | --- | --- | --- | --- |
| Provider-facing EHR APIs [Q45_1] | ☐ | ☐ | ☐ | ☐ | ☐ |
| Patient-facing EHR APIs [Q45_2] | ☐ | ☐ | ☐ | ☐ | ☐ |
| Patient-facing Payer APIs [Q45_3] | ☐ | ☐ | ☐ | ☐ | ☐ |
| Payer-to-Payer APIs [Q45_4] | ☐ | ☐ | ☐ | ☐ | ☐ |

To what extent is each of the following making it easier to use APIs to integrate with commercial EHRs or payers: *Hover mouse over bolded text for definitions.*

|  | **To a great extent** | **Moderately** | **Minimally** | **Not at all** | **Don’t know** |
| --- | --- | --- | --- | --- | --- |
| 21^st^ Century Cures Act: API regulations [Q46_1] | ☐ | ☐ | ☐ | ☐ | ☐ |
| 21^st^ Century Cures Act: Information Blocking regulations [Q46_2] | ☐ | ☐ | ☐ | ☐ | ☐ |
| Centers for Medicare & Medicaid Services Patient Access Rule [Q46_3] | ☐ | ☐ | ☐ | ☐ | ☐ |
| Centers for Medicare & Medicaid: **Blue Button 2.0** [Q46_4] | ☐ | ☐ | ☐ | ☐ | ☐ |
| Trusted Exchange Framework and Common Agreement (TEFCA) [Q46_5] | ☐ | ☐ | ☐ | ☐ | ☐ |
| HL7 FHIR Accelerators (CARIN, Argonaut, DaVinci, Gravity, Helios) [Q46_6] | ☐ | ☐ | ☐ | ☐ | ☐ |

Please share any other thoughts or comments on current or future directions for policy efforts in promoting or enforcing access to data via commercial EHR vendor or payer IT system APIs or other means. [Q47]

Continue to [concluding questions](#_CONCLUDING_QUESTIONS)

### CONCLUDING QUESTIONS

In responding to this survey, please indicate the roles/perspectives of individuals involved:

CEO/General Executive [Q48_1]

Product [Q48_2]

Engineering/Development [Q48_3]

Other: [Q48_4] please list       [Q48_4_TEXT]

We will produce a publicly available report detailing the aggregated survey findings. To promote the work you do, we plan to feature the names of the companies that responded to the survey on the report (company name only, not individual survey responses). Would you like your company's name to appear on this report? [Q49]

Yes

No

**Glossary of Terms**

Health Care Provider:

- A doctor, hospital, physician group, clinic, pharmacy, or other entity that provides health treatment or services in the regular course of business.

Covered Entity:

- A health care provider such as a doctor, hospital, physician group, clinic, or pharmacy, and others that provide health treatment or services and that file health claims or prepare other insurance transactions electronically; or
- A health plan; or
- A health care clearinghouse

Business Associate of a Covered Entity:

- A business associate is a person or entity that performs certain functions or actions that involve the use or disclosure of individually identifiable health information on behalf of, or in service to, a covered entity. Some examples include: Electronic medical record vendors, medical billing companies, document storage or disposal companies, and data analysis service providers.
- Any organization that provides data transmission services to a covered entity and that requires access to the individually identifiable health information it transmits on a routine basis is considered a business associate. An example of this type of business associate is a health information exchange organization.

Federally regulated API:

Regulation implementing a standard based approach to APIs that all vendors have to follow

US Core Data for Interoperability:

- A standardized set of health data classes and constituent data elements for nationwide, interoperable health information exchange

Blue Button 2.0:

- Blue Button 2.0 is a standards-based application programming interface (API) that delivers Medicare Part A, B, and D data for over 60 million people with Medicare. Blue Button 2.0 allows you to share your data with third-party applications, doctors, research programs, and more. It also gives beneficiaries and their caregivers more options and control over your claims data.

**APPENDIX C: List of survey respondents that consented to their company name being published**

1up.health

AIDA Healthcare

Ada

Andaman7

Andor Health

Answers Now

Astarte Medical Partners

Aunt Bertha

Azara Healthcare

BehaVR

b.well connected health

CareEvolution

Chadis

Clear Triage

Cliexa

Curai Health

Curation Health

Customer Evolution

Deep Scribe

Diagnostic IQ

Doc Buddy

Doc Response

E Visit

EBSCO

Edetek

Elimu Informatics

Esvyda

Evidence Care

Gemini Health

Getwell Loop

Grinformatics

Gyant

HAPCO

Harmony Healthcare It

Health Chain LLC

Health Data Analytics Institute

Health Gorilla

Health Hive

Health Vector

HealthTap

Healthjump

Humetrix

Hyro

I Local Box

I Treat Md

Inferscience

Innovaccer

Intellicure, LLC

Intelligent Medical Objects

Irody

Junum

Keona Health

Logi Coy

M Tuitive

MedActionPlan (CareDx)

Medisolv

Mend

Midmark

Mobius Md

Moxe Health

My Meds

Mytonomy

Nubo Health

Oh Md

Om1

One Record LLC

Opargo

Open Clinica

Oshi Health

PaceMate

Painscored

Patient Link

Patientory

Portable EMR Solutions

Premier Inc

Providertech

Qure4U

Redivus

Repisodic

Rimidi

Rx Studio

Seamless MD

Seizure Tracker LLC

Sphinx Medical Technologies

Stanson Health

Talis Clinical

Tci Software

Technosoft Healthcare Solutions

Teckel Medical Sl

The Commons Project Foundation

Thirty Madison

Truentity Corporation

Trustedi10

Unite Us

Vim

Visual Dx

Vizient Inc.

Wellsheet

WoundVision

Xealth
